# Onset and Persistence of NSSI Patterns and risk of Suicide Reattempt: Results from the SURVIVE Study

**DOI:** 10.1192/j.eurpsy.2026.10692

**Published:** 2026-07-31

**Authors:** M. Arqueros, E. Suarez-Soto, M. Diaz-Marsa, A. De La Torre-Luque, A. Cebria, M. Elices, M.-A. Boti, P. Lopez-Peña, A. Palao-Terrero, M. Ruiz-Veguilla, A. Garcia-Fernandez, T. SURVIVE Consortium, V. Perez-Sola

**Affiliations:** 1 Complutense University of Madrid; 2 San Carlos Clinic Hospital, Madrid; 3 Parc Tauli Hospital, Sabadell; 4 Mar University Hospital; 5 Clinic Hospital, Barcelona; 6 Araba Vitoria University Hospital, Vitoria; 7 La Paz University Hospital, Madrid; 8 Virgen del Rocio University Hospital, Seville; 9 University of Oviedo, Oviedo, Spain

## Abstract

**Introduction:**

After a suicide attempt, reattempt risk is concentrated in the first year, yet how post-attempt non-suicidal self-injury (NSSI) patterns relate to the timing of reattempts remains unclear. Hazard-based models can quantify this association.

**Objectives:**

To evaluate whether NSSI pattern membership predicts time to suicide reattempt over 12 months in a multivariable Cox model.

**Methods:**

Adults with a recent suicide attempt (n=685) were assessed ≤15 days post-attempt and followed for 12 months. NSSI pattern (reference=NN: No→No) was coded as Onset (No→Yes), Remission (Yes→No), or Persistence (Yes→Yes). The initial covariates were age, sex, number of prior attempts, BIS-11 subscales, ACSS-FAD, MINI diagnoses (count), BSI-GSI, reasons for ideation, and NSSI pattern. Backward elimination by Akaike’s Information Criterion (AIC) yielded the final model. We report hazard ratios (HR) with 95% CIs.

**Results:**

The final Cox model (Figure 1) retained age, number of prior attempts, reasons for ideation (reference=External), and NSSI pattern (*AIC*=833.7; *χ²*(7)=22.26, *p*<.002; concordance=.632). Older age was associated with lower hazard (*HR*=0.98, 95% CI [0.96, 0.99]); more prior attempts with higher hazard (*HR*=1.02, 95% CI [1.01, 1.04]). Relative to NN, Remission (*HR=*0.56, 95% CI [0.32, 0.96]) and Persistence (*HR*=0.58, 95% CI [0.35, 0.96]) were associated with reduced 12-month hazard; Onset did not differ significantly.

**Image 1:**
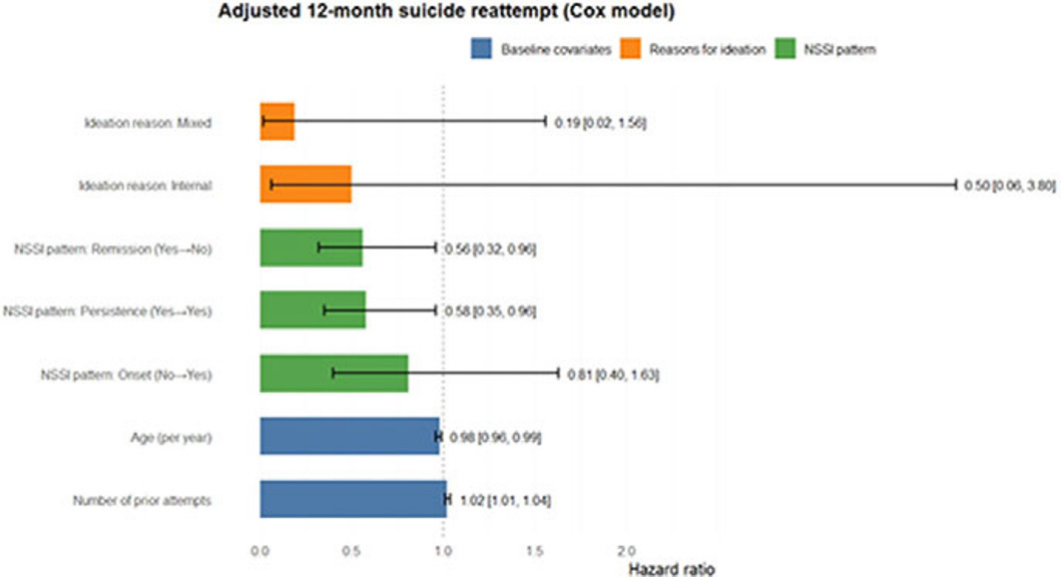
Image 1: Long description.

**Conclusions:**

After a suicide attempt, NSSI pattern membership provides prognostic information beyond age and attempt history. Compared with NN, Remission and Persistence were linked to lower 12-month reattempt hazard, whereas Onset was not. Tracking NSSI patterns may refine post-discharge risk stratification and follow-up planning.

**Disclosure of Interest:**

None Declared

## Long descriptions

### Image 1: Long description

A horizontal bar chart titled Adjusted 12-month suicide reattempt (Cox model).

Legend at the top:

- Blue: Baseline covariates

- Orange: Reasons for ideation

- Green: N S S I pattern

The x-axis represents Hazard ratio with values 0.0, 0.5, 1.0, 1.5, and 2.0. A vertical dashed line is positioned at the hazard ratio of 1.0.

Data rows from top to bottom:

1. Ideation reason: Mixed (Orange bar). Hazard ratio 0.19 [0.02, 1.56].

2. Ideation reason: Internal (Orange bar). Hazard ratio 0.50 [0.06, 3.80].

3. N S S I pattern: Remission (Yes to No) (Green bar). Hazard ratio 0.56 [0.32, 0.96].

4. N S S I pattern: Persistence (Yes to Yes) (Green bar). Hazard ratio 0.58 [0.35, 0.96].

5. N S S I pattern: Onset (No to Yes) (Green bar). Hazard ratio 0.81 [0.40, 1.63].

6. Age (per year) (Blue bar). Hazard ratio 0.98 [0.96, 0.99].

7. Number of prior attempts (Blue bar). Hazard ratio 1.02 [1.01, 1.04].

Each bar includes a horizontal error bar representing the confidence interval. The bars for Remission, Persistence, and Age are entirely to the left of the 1.0 line, while Number of prior attempts is slightly to the right.

Navigate back to Image 1:.

